# Transauricular balloon angioplasty in rabbit thoracic aorta: a novel model of experimental restenosis

**DOI:** 10.1186/1476-511X-13-33

**Published:** 2014-02-15

**Authors:** Ioanna Koniari, Efstratios Apostolakis, Athanasios Diamantopoulos, Helen Papadaki, Evangelia Papadimitriou, Evangelia Poimenidi, Dimitrios Karnabatidis, Anna Karahaliou, Lena Costaridou, Apostolos Papalois, Dimitrios Siablis, Dimitrios Dougenis, Dimitrios Alexopoulos

**Affiliations:** 1 Cardiothoracic Surgery Department, University Hospital of Patras, Rion Patras zip 25500, Greece; 2 Cardiothoracic Surgery Department of Ioannina University Hospital, Ioannina, Greece; 3 Department of Diagnostic and Interventional Radiology, Patras University Hospital, School of Medicine, Rion Patras, Greece; 4 Department of Anatomy, School of Medicine, University of Patras, Patras, Greece; 5 Laboratory of Molecular Pharmacology, Department of Pharmacy, University of Patras, Patras, Greece; 6 Department of Medical Physics, School of Medicine, University of Patras, Patras, Greece; 7 Experimental-Research Center ELPEN Pharmaceuticals, Athens, Greece; 8 Cardiology Department of Patras University Hospital, Rion Patras, Greece

**Keywords:** Atherosclerosis, Restenosis, Hypercholesterolemic diet, Balloon angioplasty, Oxidative stress, Remodelling, Intima/media ratio

## Abstract

**Background:**

The aim of this study was to demonstrate a percutaneous transauricular method of balloon angioplasty in high-cholesterol fed rabbits, as an innovative atherosclerosis model.

**Methods:**

Twenty male New Zealand rabbits were randomly divided into two groups of ten animals, as follows: atherogenic diet plus balloon angioplasty (group A) and atherogenic diet alone (group B). Balloon angioplasty was performed in the descending thoracic aorta through percutaneous catheterization of the auricular artery. Eight additional animals fed regular diet were served as long term control. At the end of 9 week period, rabbits were euthanized and thoracic aortas were isolated for histological, immunohistochemical and biochemical analysis.

**Results:**

Atherogenic diet induced severe hypercholesterolemia in both group A and B (2802 ± 188.59 and 4423 ± 493.39 mg/dl respectively) compared to the control animals (55.5 ± 11.82 mg/dl; P < 0.001). Group A atherosclerotic lesions appeared to be more advanced histologically (20% type IV and 80% type V) compared to group B lesions (50% type III and 50% type IV). Group A compared to group B atherosclerotic lesions demonstrated similar percentage of macrophages (79.5 ± 9.56% versus 84 ± 12.2%; P = 0.869), more smooth muscle cells (61 ± 14.10% versus 40.5 ± 17.07; P = 0.027), increased intima/media ratio (1.20 ± 0.50 versus 0.62 ± 0.13; P = 0.015) despite the similar degree of intimal hyperplasia (9768 ± 1826.79 μm^2^ versus 12205 ± 8789.23 μm^2^; P = 0.796), and further significant lumen deterioration (23722 ± 4508.11 versus 41967 ± 20344.61 μm^2^; P = 0.05) and total vessel area reduction (42350 ± 5819.70 versus 73190 ± 38902.79 μm^2^; P = 0.022). Group A and B animals revealed similar nitrated protein percentage (P = NS), but significantly higher protein nitration compared to control group (P < 0.01; P < 0.01, respectively). No deaths or systemic complications were reported.

**Conclusion:**

Transauricular balloon angioplasty constitutes a safe, minimally invasive and highly successful model of induced atherosclerosis in hyperlipidaemic rabbits.

## Background

Hemodynamic strain promotes the development of atherosclerosis, while dietary induced atherosclerosis is accelerated and enhanced where mechanical injury has been performed experimentally [1]. Consequently, the fact that endothelial injury and increased tendency toward atherosclerotic changes are localized in the same regions constitutes the basis for the response to injury hypothesis for atherosclerosis [2].

Restenosis, a common complication after angioplasty represents the arterial wall’s healing response to mechanical injury and comprises two main processes, neointimal hyperplasia and vascular remodeling [3-6].

Although the primary stimulus for restenosis is the mechanical injury of balloon dilation to the vessel wall, a dominant risk factor for the spontaneous development of occlusive coronary disease is hypercholesterolemia. Notably, the physical injury caused by balloon dilation induces intimal hyperplasia independent of blood cholesterol levels in angioplasty induced atherosclerotic lesions [7].

Traditionally, in experimental atherosclerosis models, intraarterial access in an animal is achieved through femoral artery surgical cut-down. This technique however, may occasionally be followed by severe complications involving bleeding, thrombosis, arterial occlusion and local or systemic infections. The aim of this study is to describe a safe, non-surgical percutaneous method of transauricular endovascular access and further balloon angioplasty performance in the thoracic aorta of high-cholesterol fed rabbits, as a novel alternative model of experimental restenosis.

## Results

### Transauricular arterial access for balloon injury of thoracic aorta

Percutaneous catheterization of the auricular artery and further balloon injury of the descending thoracic aorta was performed successfully in all rabbits of group A (10 of 10 animals), with no complication being noted during angiographic examination. In one case, puncture of the rabbit auricular artery resulted in severe vasospasm, with subsequent inability to infuse contrast medium and insert the guide wire. In this rabbit, vascular access was achieved through the contra lateral central auricular artery. The recovery of all group A rabbits was normal without any local or systemic complications. No clinical signs of hematoma or local infection were identified. There were no deaths after the intervention and the following 8 week period.

After the transauricular injury of descending aorta, the punctured auricular artery was peripherally destroyed and could not be re- accessed.

### Blood chemistry

At the end of the 9-week period mean TC, TG, and HDL levels (mg/dl) increased significantly in both group A and group B hypercholesterolemic animals compared to control group (P < 0.001; Table 1). A statistically significant increase in TC, TG and HDL levels (P < 0.001; P = 0.001; P = 0.03, respectively) was observed in group B compared to group A animals.

**Table 1:** Blood assays of control, injured and non- injured hyperlipidemic rabbits

**Blood assays**	**Group A**		**Group B**		**Control**	
	**5 weeks**	**9 weeks**	**5 weeks**	**9 weeks**	**5 weeks**	**9 weeks**
**TC**	2005 ± 207.20	2802 ± 188.59^a^	4121 ± 414,99	4423 ± 493.39^b,c^	35 ± 7.25	55.5 ± 11.82
**TG**	197 ± 32.30	324 ± 33.73^a^	381 ± 54.56	502 ± 96.24^b,c^	38 ± 2	43.8 ± 9.66
**HDL**	295 ± 53.98	384 ± 26.29^a^	383 ± 40.49	412 ± 15.40^b,d^	21.8 ± 3.60	34.5 ± 2.88
**SGPT**	31 ± 7.10	38 ± 6.78	47 ± 5.53	54 ± 5.57	32.5 ± 8	39.7 ± 6
**g-GT**	5 ± 2.15	7 ± 1.43	6 ± 2.05	7 ± 1.58	4.25 ± 0.89	5.25 ± 0.89
**Creatinine**	1.05 ± 0.14	1.03 ± 0.13	0.79 ± 0.11	0.89 ± 0.13	0.78 ± 0.46	0.93 ± 0.18

*Baseline biochemical parameters of all groups were into the normal range.

^a^P < 0.001, unpaired *t*-test vs Control.

^b^P < 0.001,unpaired t- test vs Control.

^c^P ≤0.001, unpaired *t*-test vs Group A.

^d^P < 0.05, unpaired *t*-test vs Group A.

Noteworthily, at the end of 9-week period, both high-cholesterol fed rabbits subjected to balloon injury and non-injured hypercholesterolemic rabbits, had normal renal and liver function, as levels of creatinine and hepatic enzymes remained within normal range (Table 1). Finally, there was no statistically significant difference in body weight between control, group A and group B rabbits (3705 ± 140.34 g, 3625 ±88,64 g and and 3600 ± 92.58 g, respectively; P = 0.137).

### Histological evaluation of atherosclerosis

Atherogenic diet resulted in the development of significant atherosclerotic lesions in all group A and group B animals compared to the control group, that revealed no visible atherosclerotic lesions (p < 0.001). Advanced type IV (atheroma; n = 2 animals) and type V (fibroatheroma: n = 8 animals) with or without calcification (Vb or Va) atherosclerotic lesions were observed in injured thoracic aortas of group A rabbits. While, intermediate type III (n = 5 animals) and advanced type IV (n = 5 animals) atherosclerotic lesions were present in non-injured thoracic aortas of group B animals. In conclusion, balloon angioplasty in descending thoracic aortas induced statistically significant atherosclerotic lesions in group A compared to group B animals (P < 0.001).

**Figure 1:**
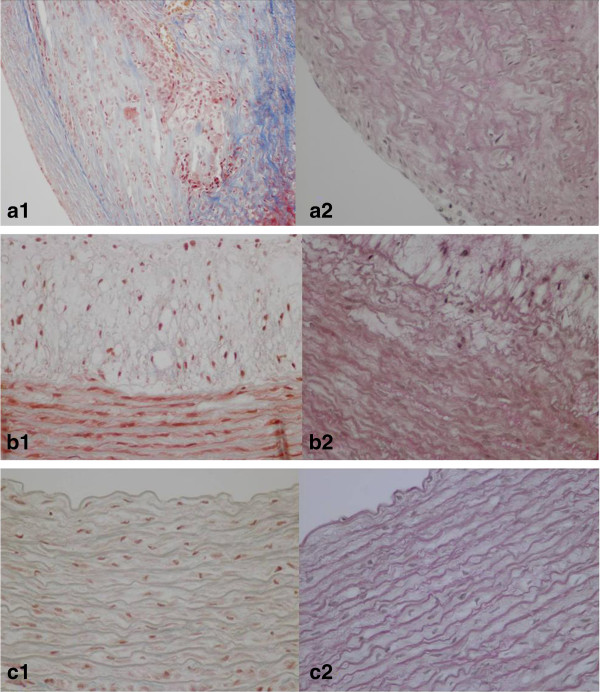
**Masson’s trichrome and Van Gieson staining in descending thoracic aortas. a1**. Great amount of collagen tissue deposition, and **a2**. Severe elastic fiber disruption and disorientation in balloon injured atherosclerotic thoracic aortas. **b1**. Slight collagen tissue increment and **b2**. Focal fragmentation and disorientation of elastic tissue in non-injured atherosclerotic thoracic aortas. **c1**. Absence of fibrosis, and **c2**. normal elastic fiber orientation in control thoracic aortas.

Masson and Van Gieson staining revealed adequate amount of collagen tissue deposition in the tunica media and intima (Figure 1a1), as well as prominent disruption of elastic fibers in both the internal elastic lamina and the tunica media of injured atherosclerotic aortas (Figure 1a2). While, there was a slight deposition of collagen tissue in the tunica media and intima (Figure 1b1), accompanied by disruption of elastic fibers in internal elastic lamina of non- injured atherosclerotic aortas (Figure 1b2). No elastic fiber disruption or collagen deposition was observed in aortas of control rabbits (Figure 1c).

Interestingly, the severity of the aortic atherosclerosis as defined by histological analysis was in accordance with the elevated serum cholesterol levels and the mechanical injury caused by balloon dilatation in spontaneous and angioplasty induced lesions, respectively. Additionally, mechanical injury in conjunction with hypercholesterolemia resulted in more prominent atherosclerotic lesions than atherogenic diet alone.

### Immunohistochemical study

**Figure 2:**
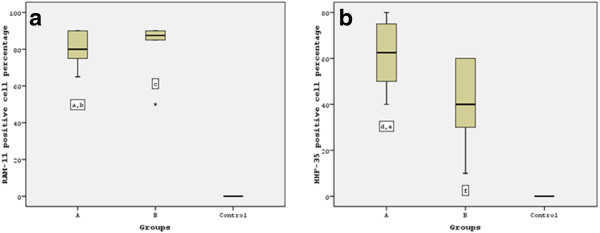
**RAM-11 and HHF-35 positive cell distribution in three groups. a**. No significant difference in RAM-11 cell percentage was observed between angioplasty induced and spontaneous atherosclerotic lesions: a P = non significant, Bonferroni’s post hoc ANOVA analysis vs Group B; b P < 0.001, Bonferroni’s post hoc ANOVA analysis vs contol; c P < 0.001, Bonferroni’s post hoc ANOVA analysis vs control. **b**. Significant increase in HHF-35 cells was noticed in angioplasty induced atherosclerotic lesions: d P < 0.05, Tamhane’s post hoc ANOVA analysis vs Group B; e P < 0.001, Tamhane’s post hoc ANOVA analysis vs control; f P < 0.001, Tamhane’s post hoc ANOVA analysis vs control.

**Figure 3:**
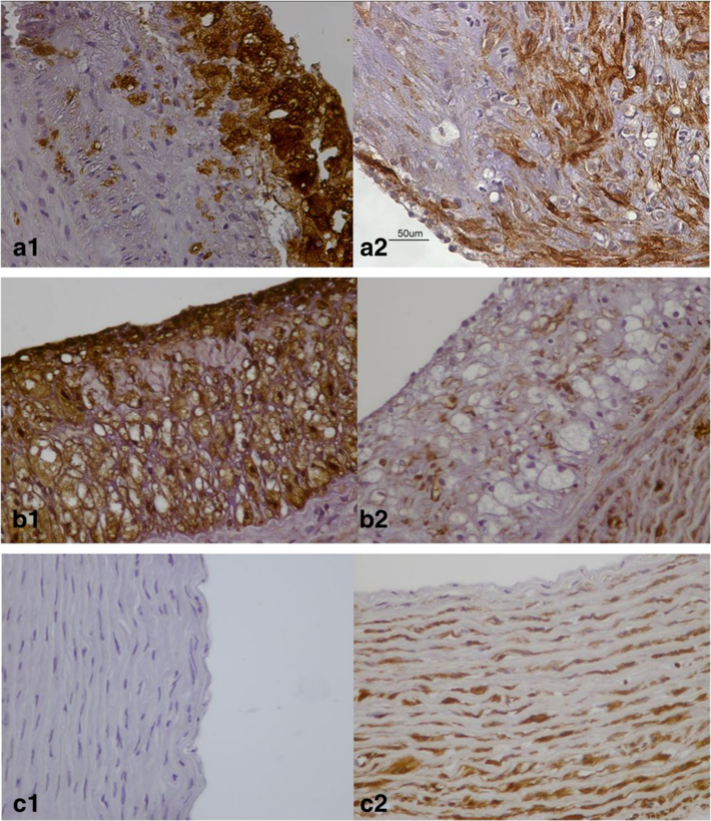
**Ram-11 and HHF-35 staining in thoracic aortas. a1**. Plethora RAM-11 positive foam cells, and **a2**. significant amounts of positive for a-actin SMCs are observed in angioplasty induced atherosclerotic lesions. **b1**. Significant foam cell formation, and **b2**. several a-actin positive SMCs between foam cells in spontaneous atherosclerotic lesions. **c1**. Lack of foam cell deposition, and **c2**. Normal presence of HHF-35 positive SMCs in the media of control aortas.

The atherogenic diet was associated with a significant increase in lipid deposition and foam cell formation, indicated by the increase in RAM-11 immunoreactivity in group A and B animals. The aortas of group A rabbits were mainly strongly positive (n = 8 animals) for RAM-11 staining, except two cases that were moderately positive. Similarly, almost all the aortas of group B rabbits revealed strongly positive (n = 9 animals) RAM-11 immunoreactivity. It is prominent that the percentage of RAM-11 positive cells had no significant difference between spontaneous and angioplasty- induced atherosclerotic lesions (79.5 ± 9.56% versus 84 ± 12,2% respectively; P = 0.869), as shown in Figure 2. Also, the relative number of macrophages was associated with the severity of atherosclerotic lesions, as demonstrated in Figure 3a1 and 3b1.

HHF-35 immunostaining for α-actin, demonstrated that angioplasty- induced atherosclerotic lesions of group A were mainly moderate positive (n = 7 animals) or strongly positive in three cases (Figure 3a2). Whereas, spontaneous atherosclerotic lesions of group B, contained either mild (n = 4 animals) or moderate (n = 6 animals) numbers of HHF-35 immunopositive SMCs (Figure 3b2). There was a statistically significant difference in SMCs percentage between angioplasty-induced and spontaneous atherosclerotic lesions (61 ± 14.10% versus 40.5 ± 17.07% respectively; P = 0.027). Notably, the prominent increment of HHF-35 positive cells in angioplasty- induced lesions, reflects the key role of SMCs in restenosis after balloon angioplasty.

Control animals demonstrated no RAM-11 or HHF-35 staining (Figure 3c1, 3c2), revealing significant difference regarding the amount of foam cells and SMCs compared to group A and group B animals, respectively (P <0.001).

### Morphometric analysis

**Figure 4:**
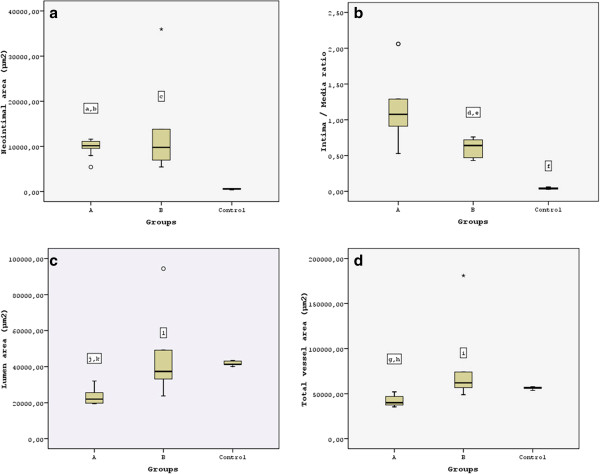
**Comparison of neointimal area, intima/media ratio, lumen area and total vessel area between groups. a**. Atherogenic diet and balloon angioplasty induced significant intimal hyperplasia in hyperlipidaemic rabbits: a P = non significant, Tamhane’s post hoc ANOVA analysis vs Group B; b P < 0.001, Tamhane’s post hoc ANOVA analysis vs control; c P < 0.05, Tamhane’s post hoc ANOVA analysis vs control. **b**. A significant increase in intima/media ratio was observed in angioplasty induced atherosclerotic lesions: d P < 0.05, Tamhane’s post hoc ANOVA analysis vs Group A; e P < 0.001, Tamhane’s post hoc ANOVA analysis vs Control; f P < 0.001, Tamhane’s post hoc ANOVA analysis vs Group A. **c**. Angioplasty resulted in a significant reduction in lumen of injured thoracic aortas: j P = 0.05, Tamhane’s post hoc ANOVA analysis vs Group B; k P < 0.001, Tamhane’s post hoc ANOVA analysis vs control; l P = non significant, Tamhane’s post hoc ANOVA analysis vs control. **d**. Injured thoracic aortas demonstrated a significant decrease in total vessel area consistent with constrictive remodeling occurrence: g P < 0.05, Bonferroni’s post hoc ANOVA analysis vs Group B; h P = non significant, Bonferroni’s post hoc ANOVA analysis vs control; i P = non significant, Bonferroni’s post hoc ANOVA analysis vs control.

Atherosclerotic lesions were not present in the thoracic aorta of control animals. Atherogenic diet induced significant intimal hyperplasia in spontaneous atherosclerotic lesions in group B compared to control group (12205 ±8789.23 μm^2^ versus 555 ± 134.75 μm^2^; P < 0.001), (Figure 4a). Similarly, balloon angioplasty induced significant intimal hyperplasia in hyperlipidemic animals of group A compared to animals maintained on control diet (9768 ± 1826.79 μm^2^ versus 555 ± 134.75 μm^2^; P < 0.001). No significant difference was observed in intimal hyperpasia between angioplasty- induced and spontaneous atherosclerotic lesions in group A and B, respectively (9768 ± 1826.79 μm^2^ versus 12205 ± 8789.23 μm^2^; P = 0.796), (Figure 4a). However, angioplasty induced atherosclerotic lesions demonstrated a significant increase in intima/media ratio compared to spontaneous atherosclerotic lesions (1.20 ± 0.50 versus 0.62 ± 0.13, in groups A and B respectively; P = 0.015). Both angioplasty-induced and spontaneous atherosclerotic lesions revealed a significant increase in intima/media ratio compared to the control animals (P <0.001), (Figure 4b). In addition, balloon dilatation resulted in a significant lumen deterioration in angioplasty induced lesions compared to spontaneous atherosclerotic lesions (23722 ± 4508.11 versus 41967 ± 20344.61 μm^2^; P = 0.05). Thus, there was a significant reduction in lumen of angioplasty induced lesions compared to the respective of the control group (23722 ± 4508.11 versus 41828 ± 1213.53 μm^2^; P < 0.001), whereas there was no difference in lumen area between group B and control animals (41967 ± 20344.61 μm^2^ versus 41828 ± 1213.53 μm^2^; P = 1), (Figure 4c). Finally, a significant reduction in total vessel area was observed between injured and uninjured thoracic aortas in group A and B, respectively (42350 ± 5819.70 versus 73190 ± 38902.79 μm^2^; P = 0.022), whereas there was observed a non-significant decrease in total vessel area between injured and control thoracic aortas (42350 ± 5819.70 versus 56298 ± 1346.73 μm^2^; P = 0.672), (Figure 4d). A non-significant increase was observed in total vessel area between hypercholesterolemic and control rabbits (73190 ± 38902.79 μm^2^ versus 56298 ± 1346.73 μm^2^; P = 0.43).

### Biochemical analysis

**Figure 5:**
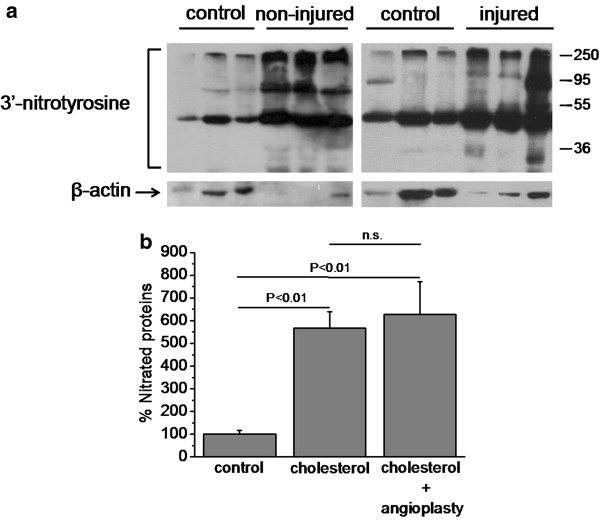
**Tyrosine nitration of proteins is not increased by the transauricular balloon angioplasty. a**. Western Blot analysis for 3′-nitrotyrosine (upper panel) and β-actin (lower panel) in total protein lysates of descending aortas. **b**. The protein amounts were quantified by densitometric analysis of the corresponding bands and the ratio of nitrated proteins/β-actin was calculated for each lane. Results are expressed as mean ± S.E.M. of the percentage change of the amounts of 3′-nitrotyrosine compared with control animals. n.s, not statistically significant.

The effect of the proposed injury model on protein tyrosine nitration was studied by measuring protein nitrotyrosine levels on aorta samples derived from control and animals fed with high cholesterol diet, injured and non-injured. High cholesterol diet significantly increased protein nitration levels, while transauricular ballon angioplasty did not cause any further increase (Figure 5).

## Discussion

We used a non-surgical, minimally invasive technique to perform balloon injury in rabbit thoracic aortas by percutaneous catheterization of the auricular artery. In experimental restenosis models, catheterizations are traditionally performed after surgical cutdown of the femoral or carotid arteries and followed by subsequent balloon injury in femoral, iliac, coronary, carotid arteries and abdominal or thoracic aorta, respectively. The peripheral vessels of the rabbit are fragile, while surgical cut-down and catheterization are associated with long procedural time periods and deep general anesthesia with intubation [8]. Moreover, a surgically accessed vessel is finally ligated, which frequently renders it vulnerable to iatrogenic trauma or even thrombosis. On the contrary, our alternative catheterization method is simple, rapid, safe and easily reproducible, as it is takes advantage of favourable auricular vascular anatomy of the rabbit. The major benefits of transauricular catheterization technique comprise the acceleration of endovascular access, the avoidance of surgical wounds, and, significantly, the preservation of valuable femoral and cervical vessels. In addition, animals experience less pain, bleeding complications, wound infections, while dissociative anaesthesia is sufficient.

Generally, the technique offers the advantages of percutaneous minimally invasive procedures, characterized by reduced morbidity and mortality as well as minimal distortion of normal anatomy and physiology. A minor disadvantage of transauricular catheterization method constitutes the peripheral auricular artery impairment that could be attributed to the cutting of the dermis along the course of the guide wire and further multiple dilatations so as to be achieved the insertion of sheaths.

In our model of induced atherosclerosis, angioplasty –induced atherosclerotic lesions appeared to be significantly more severe and advanced (type V) compared to spontaneous (type III or type IV) lesions, confirming studies with balloon injury through other approaches. It has been noticed that in the absence of hypercholesterolemia, angioplasty- induced lesions regress and resolve spontaneously [9], while, in the presence of hypercholesterolemia these lesions not only sustain but progress to larger lesions. Also, plaque size at sites of spontaneous lesions has been reported to increase with increasing blood cholesterol levels [7]. Similarly, in our study, both spontaneous and angioplasty induced atherosclerotic lesions revealed significant intimal hyperplasia in uninjured and balloon injured rabbits respectively, after 9 weeks of 4% high cholesterol diet.

Another difference in pathogenesis between spontaneous and angioplasty-induced atherosclerotic lesions consists in cell type population. Especially, SMCs have been found to be abundant in the intima of balloon- injured aortas, whereas foam cell-derived macrophages have been recognized as the predominant cell type in the intima of undamaged aortas [10]. In fact, we demonstrated that the percentage of macrophages (RAM-11 positive cells) revealed no difference between spontaneous and angioplasty- induced lesions and was in accordance with the histological stage of the respective lesions. Whereas, the percentage of SMCs (HHF-35 positive cells) revealed a statistically significant increase in angioplasty-induced lesions compared to spontaneous lesions of uninjured aortas, similarly to previous studies [11,12].

Additionally, angioplasty induced lesions demonstrated a significant increase in intima/media ratio compared to spontaneous atherosclerotic lesions, despite the similar degree of intimal hyperplasia. Furthermore, the increased intima/media ratio was accompanied with significant lumen deterioration and total vessel reduction in angioplasty induced lesions, reflecting the artery’s shrinkage or constrictive remodeling. Indeed, the accumulation of SMCs in combination with ECM reorganization that characterized by increased collagen deposition, contributes to intimal thickening and negative remodeling, leading in restenosis after balloon angioplasty.

It should be noticed the safety of both transauricular balloon angioplasty and atherogenic diet with regard to the induction and progression of atherosclerosis. Especially, we used a non-commercial and easy manufactured atherogenic diet consisting of standard rabbit chow enriched with 4% cholesterol [13], without any additional atherogenic components, such as palm oil [14], peanut oil [15], high fat coconut oil [16], high fat corn oil [17], and lard combined with either yolk powder or peanut oil [18,19]. Interestingly, the normal renal and liver function of balloon injured hyperlipidaemic animals confirms the absence of complications with percutaneous transauricular angioplasty, suggesting a safe alternative non-commercial atherogenic diet together with a minimally invasive restenosis model.

Generally, hypercholesterolemia has been known to induce the development of atherosclerotic lesions both in humans and animal models, while the cellular basis for this action has been largely attributed to the formation of oxidized LDL (oxLDL) and oxidative injury to endothelium [20]. It has been reported that the oxidative modification of LDL correlated with reduction of endothelial nitric oxide synthase (eNOS) expression [21] or modulation of eNOS activity and free radical bioavailability [22], resulting in peroxynitrite and 3′-nitrotyrosine formation. Interestingly, application of the transauricular balloon injury model did not further increase protein nitration observed in hyperchoresterolemic animals, suggesting that balloon angioplasty does not cause peroxynitrite-mediated oxidative stress.

## Conclusions

This study in high-cholesterol fed rabbits introduces transauricular balloon angioplasty as a novel model of induced- atherosclerosis and vascular restenosis. Therefore, transauricular angioplasty constitutes a safe, rapid, minimally invasive, highly successful as well as easily reproducible restenosis model.

## Methods

### Animal model

The study was conducted in accordance to the Institutional “Guide for the Care and Use of Laboratory Animals” and was approved by the Institutional Animal Care and Use Committee of the West Greece Prefecture. All experiments were performed in the Animal House of the Medical School. Twenty eight male New Zealand White rabbits, weighing 2.5–3 kg, were housed individually at 20 ± 3°C with a 12-h: 12-h light/dark cycle and with free access to water. All rabbits were allowed one week, feeding on regular rabbit chow, to acclimate to their environment. Eight animals were fed regular rabbit chow until the end of the study, serving as long term control group (C; n = 8). After the week of acclimation, atherogenic diet consisting of regular rabbit chow supplemented with 4% cholesterol (ELPEN Pharmaceutical, Athens, Greece) was initiated. Then twenty rabbits were randomly divided into two groups of ten animals, as following: atherogenic diet plus balloon angioplasty (group A) and atherogenic diet alone (group B). Seven days later, rabbits of group A were anesthetized with ketamine (50 mg/kg) plus xylazine (10 mg/kg) intramuscularly, and subjected to balloon injury of the descending thoracic aorta through percutaneous catheterization of the auricular artery [8]. At the end of the intervention, antibiotic prophylaxis with cephalosporin was administered (750 mg cefuroxime intramuscularly; Zinacef; GlaxoSmithKline, Research Triangle Park, NC). The animals were allowed to recover and were maintained on 4% high- cholesterol diet for additional 8 weeks. At the end of this period, all animals were subsequently anesthetized using the above mentioned regimens (ketamine plus xylazine), and euthanized by intravenous injection of a saturated KCl solution. The descending aortas were exposed, isolated and harvested for histological, immunohistochemical, and biochemical analysis. Rabbit feeding was restricted to 120 g/day. Blood samples were collected every 4 weeks. The general condition of the rabbits was observed daily. Body weights were measured every 4 weeks.

### Transauricular approach

The detailed technique of envovascular transauricular access has been previously described [8]. Here, we describe the balloon angioplasty performance after percutaneous catheterization of the rabbit auricular artery. In brief, animals were immobilized in the supine position, and both auricular dorsa (ie, backside surfaces of their ears) were shaved and scrubbed with a combination of povidone iodine and an alcohol-based solution to achieve disinfection. Rabbits were placed under a c-arm angiographic unit with ability to perform road mapping and digital subtraction angiography (Philips DVI-S angiography unit). Cardiovascular monitoring was performed with peripheral pulse oximetry.

**Figure 6:**
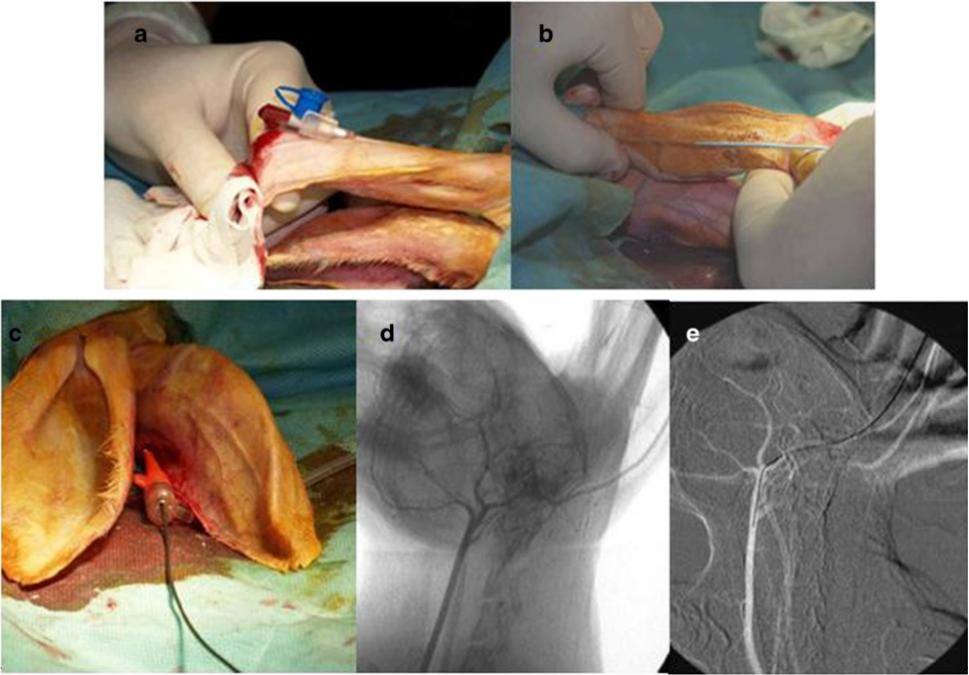
**Intraarterial transauricular access in the rabbit thoracic aorta. a**. Direct percutaneous catheterization of the central auricular artery with a 22-gauge intravenous catheter, **b**. Repeated tract dilations of the peripheral transauricular artery with the introduction of a 4-F vascular sheath, **c**. Insertion of an 6.0-mm/40- mm (Cordis) peripheral dilatation balloon catheter over the guide wire, d-e Roadmap image of the auricular artery and the common carotid artery.

The central auricular artery was the target vessel for cannulation and endovascular access of the descending thoracic aorta respectively. Initially, the auricular artery was punctured with a 22-gauge intravenous catheter (Helmflon; Helm Pharmaceuticals, Hamburg, Germany) approximately at the distal half of its subcutaneous course (Figure 6a). The needle of the catheter was removed, and 5 mL of contrast agent diluted with normal saline (1:1) was infused to obtain roadmap images of the extracranial carotid vasculature. Care was taken to aim at the distal half of the vessel, so as a second more proximal attempt might be performed in case of vasospasm or rupture [8].

**Figure 7:**
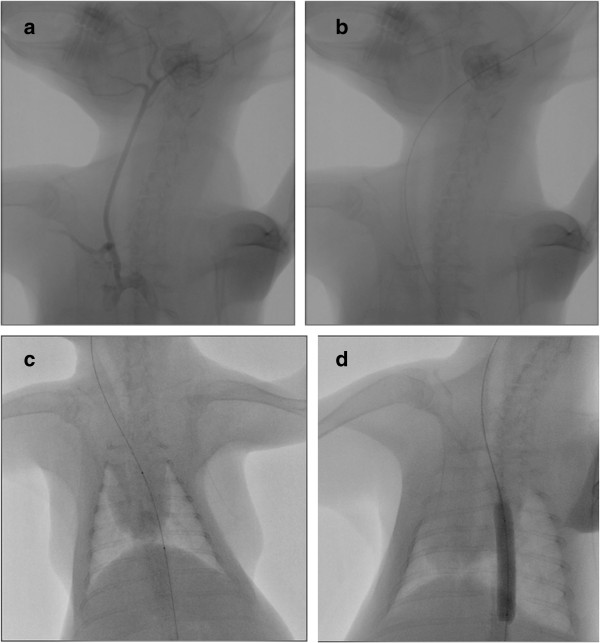
**Balloon angioplasty of descending thoracic aorta. a**. Roadmap images of the rabbit’s carotid artery and the thoracic aorta, **b**. Insertion of the guide wire into the descending thoracic aorta, **c**. Insertion of a 6.0-mm/40-mm peripheral dilatation balloon catheter over the guide wire and placement to the level of descending thoracic aorta **d**. Inflation of the balloon and injury of the descending aorta below the orifice of subclavian artery.

Secondly, a 0.018-inch guide wire (V-18 control wire; Boston Scientific, Natick, MA) was carefully advanced into the external carotid artery (Figure 6d and 6e). The guide wire was then advanced in the aortic arch and navigated into the descending thoracic aorta under fluoroscopic guidance (Figure 7). Then, the intravenous catheter was withdrawn, local anesthesia (lidocaine 1%) was applied, and a 2- to 3-cm-long incision of the dermis was performed at the point of the initial puncture along the course of the guide wire. Finally, a 4-F, 0.018- inch guide wire–compatible vascular sheath (Bolton Medical, Villers-les- Nancy, France) was advanced into the external carotid artery after serial step-by-step dilations with the sheath’s own dilator (Figure 6b), [8]. The repeated over-the wire dilations were necessary to remove the tight and narrow peripheral segment of the arterial endothelium before sheath insertion. All animals were administered an intravenous bolus of heparin (100 U/kg) after vascular access was established. When transauricular arterial access had been gained, a 6.0-mm/40- mm (Cordis, Abbott Vascular, Santa Clara, USA) peripheral dilatation balloon catheter, preinfused with heparinized saline, was inserted over the guide wire and advanced to the level of descending thoracic aorta below the orifice of subclavian artery (Figures 6c and 7). Injury of the descending aorta was performed by inflating the balloon twice to 11 atm of pressure for 45 seconds at 30 second intervals. The balloon was then removed from the animal and angiographic imaging of the descending thoracic aorta was performed in order to exclude rupture or dissection of the vessel. Antibiotic prophylaxis using 750 mg of cefuroxime was given intramuscularly. Animals were monitored during recovery and were closely checked for any signs of local hematomas and local or systemic infection.

### Biochemical assays

Total serum cholesterol (TC), triglyceride (TG), and high density lipoprotein (HDL) levels were measured. Renal and liver function were monitored using creatinine and serum hepatic enzymes (SGPT and g-GT) levels, respectively.

### Histological analysis

Balloon injured descending thoracic aortas were fixed by immersion in neutrally buffered 10% formalin, followed by dehydration and embedding in paraffin wax using standard procedures. Four-micrometer sections were obtained from each vessel at 5-mm intervals and stained with hematoxylin and eosin for histopathologic analysis. Masson’s trichrome aniline blue and Weigert Van Gieson’s elastic stains (Histoline Laboratories, Italy) were used to assay collagen components and the thickness of the intima, respectively.

Spontaneous and angioplasty induced atherosclerotic lesions were classified according to the guidelines of the American Heart Association [23].

### Immunohistochemistry

Consecutive 4-μm-thick sections from each aortic specimen were collected on Superfrost plus glass slides, deparaffinized, and rehydrated in graded alcohols. Endogenous peroxidase activity was blocked by treatment with 3% hydrogen peroxide for 15 min, followed by incubation with protein blocking solution to eliminate nonspecific binding. Immunohistochemistry was performed using monoclonal antibodies to detect macrophages (RAM 11, 1:200 dilution; Dako Corp, CA, USA), and α-actin in SMCs (HHF-35, 1:100 dilution; DAKO A/S). The Envision Plus Detection System kit (DakoCytomation, USA) and 3, 3′-diaminobenzidine (DAB) were used to visualize antibody binding, according to manufacturer’s instructions. Sections were counterstained with Harris’ hematoxylin, dehydrated, and mounted permanently. For each antibody, all tissues from the different study animals were immunostained concurrently. Negative controls were performed in all cases by omitting the primary antibodies.

Immunohistochemical staining was graded on a scale of 0 to 3 based on the percentage of immunopositive cells as follows: 0, <10% positive cells; 1 (mildly positive), 10–35% positive cells; 2 (moderately positive), 35–70% positive cells; and 3 (strongly positive), >70% positive cells.

### Morphometry

Cross sectional areas were quantified using IMAGE J software [24]. For each artery, measurements of luminal area (LA); area bounded by the internal elastic lamina (IEL; corresponding to the LA in the absence of intimal lesions); and area encircled by external elastic lamina (EEL; corresponding to overall vessel size) were performed. Neo-intimal area (IA) was determined by subtracting the LA from the area encircled by the IEL. The medial area (M) was calculated as the area encircled by EEL minus the area encircled by the IEL. Total vessel area, defined as the area encircled by the EEL, and intima/media (I/M ratio) were determined.

### Western blot analysis

Protein tyrosine nitration is one of the post-translational modifications derived from the reaction of proteins with nitrating agents, such as peroxynitrite, or the activity of myeloperoxidase and eosinophil peroxidase. Protein nitration can lead to biological function alterations and has been detected in several pathological situations, such as cancer, inflammation and atherosclerosis [25].

Descending aorta samples were homogenized in RIPA buffer and total lysates (100 μg/sample) were run on a 10% SDS-PAGE gel and transferred to Immobilon P membranes. Blocking was performed by incubating the membranes with Tris-buffered saline (TBS), pH 7.4, with 0.1% Tween (TBS-T), containing 5% nonfat dry milk. Membranes were incubated with primary antibody for 3′-nitrotyrosine (Upstate Biotechnology, #06-284) or β-actin (Santa Cruiz, #sc58673), in a dilution of 1:1,000 in TBS-T, for 16 h at 4°C under continuous agitation. The membranes were washed 3 times with TBS-T for 5 min and incubated with HRP- linked anti-rabbit IgG (1:12,500 in TBS-T, Sigma #A0545) or anti-mouse IgG (1:5,000 in TBS-T, Sigma #A3682) respectively, for 1 h at room temperature. Detection of immunoreactive bands was performed using the enhanced chemiluminescence (ECL) detection kit (Pierce Biotechnology, Rockford, IL, USA). The protein levels that corresponded to the immunoreactive bands for 3′-nitrotyrosine and β-actin were quantified using the ImagePC image analysis software (Scion Corp., Frederick, MD, USA) and the ratio of nitrated proteins/β-actin was calculated for each lane.

### Statistical analysis

SPSS for Windows (release 19. 0. 0 SPSS Inc, Chicago IL, USA) was used for continuous data analysis. All data were expressed as the mean ± standard deviation (SD). Comparison among groups was performed by ANOVA. Comparison between two specific groups was performed by the unpaired two-tailed Student’s *t*-test. A p value < 0.05 was regarded as statistically significant.

## Competing interest

The authors declare that they have no competing interests.

## Authors’ contributions

All authors participated in the design, interpretation of the studies, analysis of the data and review of the manuscript. IK, EA, AD, DK conducted the experiments; DK, DS, DD, DA and LC supplied critical reagents; IK wrote the manuscript; HP performed the histological analysis; EP and EP performed the biochemical assays; AK and IK conducted the morphometric analysis; and IK conducted the statistical analysis. All individuals who made contributions to this study are included as authors. All authors read and approved the final manuscript.
